# Feasibility of Fiber Bragg Grating and Long-Period Fiber Grating Sensors under Different Environmental Conditions

**DOI:** 10.3390/s101110105

**Published:** 2010-11-10

**Authors:** Jian-Neng Wang, Jaw-Luen Tang

**Affiliations:** 1 Department of Construction Engineering, National Yunlin University of Science and Technology, Douliou 64002, Taiwan; 2 Department of Physics, National Chung Cheng University, Chia-Yi 62102, Taiwan

**Keywords:** nondestructive evaluation (NDE), fiber Bragg grating (FBG), long-period fiber grating (LPFG), temperature, strain, liquid-level, finite element model, random walk coefficient, 07.60.Vg, 42.81.-i., 07.05.-t

## Abstract

This paper presents the feasibility of utilizing fiber Bragg grating (FBG) and long-period fiber grating (LPFG) sensors for nondestructive evaluation (NDE) of infrastructures using Portland cement concretes and asphalt mixtures for temperature, strain, and liquid-level monitoring. The use of hybrid FBG and LPFG sensors is aimed at utilizing the advantages of two kinds of fiber grating to implement NDE for monitoring strains or displacements, temperatures, and water-levels of infrastructures such as bridges, pavements, or reservoirs for under different environmental conditions. Temperature fluctuation and stability tests were examined using FBG and LPFG sensors bonded on the surface of asphalt and concrete specimens. Random walk coefficient (RWC) and bias stability (BS) were used for the first time to indicate the stability performance of fiber grating sensors. The random walk coefficients of temperature variations between FBG (or LPFG) sensor and a thermocouple were found in the range of −0.7499 °C/ $\sqrt{h}$ to −1.3548 °C/ $\sqrt{h}$. In addition, the bias stability for temperature variations, during the fluctuation and stability tests with FBG (or LPFG) sensors were within the range of 0.01 °C/h with a 15–18 h time cluster to 0.09 °C/h with a 3–4 h time cluster. This shows that the performance of FBG or LPFG sensors is comparable with that of conventional high-resolution thermocouple sensors under rugged conditions. The strain measurement for infrastructure materials was conducted using a packaged FBG sensor bonded on the surface of an asphalt specimen under indirect tensile loading conditions. A finite element modeling (FEM) was applied to compare experimental results of indirect tensile FBG strain measurements. For a comparative analysis between experiment and simulation, the FEM numerical results agreed with those from FBG strain measurements. The results of the liquid-level sensing tests show the LPFG-based sensor could discriminate five stationary liquid-levels and exhibits at least 1,050-mm liquid-level measurement capacity. Thus, the hybrid FBG and LPFG sensors reported here could benefit the NDE development and applications for infrastructure health monitoring such as strain, temperature and liquid-level measurements.

## 1. Introduction

Technologies for real-time non-destructive evaluation (NDE) of engineering structures are very important to access the performance of in-service infrastructures. It is very difficult to carry out on-line infrastructure integrity monitoring using classical NDE methods. Fiber optic sensors (FOSs) in smart structures provide a special opportunity for real-time-multiplexed monitoring of the health status of infrastructure using surface-bonded or embedded sensors. FOSs are very important sensors used in smart materials and structures because of their advantages compared with commonly used NDE technologies [1]. FOSs used for the NDE of advanced materials and structures have been demonstrated to be feasible for the measurement of material changes during fabrication, the in-service lifetime measurement of strain, temperature, and other physical perturbations, and the eventual detection of damage or material degradation [2]. Smart materials and structures provide the real possibility of structural integrity monitoring on-line, *in situ* or *in vivo*. FOSs in smart structures are an enabling technology that will allow engineers to establish a nervous or feedback system in their designs, in which the system performs structural damage monitoring and assessment, whereas it is difficult to accomplish the above tasks with common NDE technologies [3].

The advantages of fiber optic sensors include light weight, small size, geometrical versatility, immunity to electromagnetic interference (EMI), large bandwidth, environmental ruggedness, and electrical and optical multiplexing. Thus, fiber optic sensors are ideal sensors for potential smart structure and material applications. In recent years, the technology of fiber optic sensors has been applied to the field of structural monitoring, infrastructure assessment, and some industrial sectors [3–6]. The most attractive feature of fiber optic sensors is their inherent ability to serve as both the sensing element and the signal transmission medium, allowing the electronic instrumentation to be located remotely from the measurement site. This is especially useful for remote monitoring of the condition of infrastructures.

In this work, a reference dual-wavelength grating FBG and LPFGs were examined to evaluate typical infrastructure materials, such as Portland cement concretes and asphalt mixtures, for temperature, strain, and liquid-level measurements as these three physical measurements are the common measurands for infrastructure materials regarding thermal effects, mechanical response, and liquid-level monitoring. The experimental results presented here include temperature fluctuation and stability tests using both FBG and LPFG, respectively. An indirect tensile strain test using a FBG sensor as well as finite element modeling were carried out for comparative analysis. Liquid-level measurements using a sensor constructed by cascading five different wavelength LPFGs are presented. The use of hybrid FBG and LPFG sensors are shown to combine the advantages of both kinds of fiber grating to work on nondestructive evaluation of infrastructures such as bridges, pavements, and reservoirs for strains or displacements, temperatures, and water-levels under different environmental conditions. Thus, researchers or engineers could potentially use FBG and LPFG sensors with the same measurement apparatus, such as the ASE light source and optical spectrum analyzer (OSA), to monitor changes of strain, temperature, and liquid-level over time.

## 2. Fiber Grating Sensors

**Figure 1.**
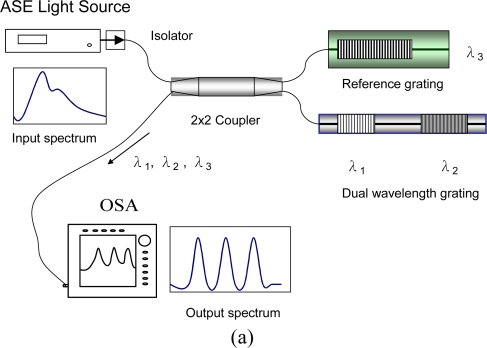

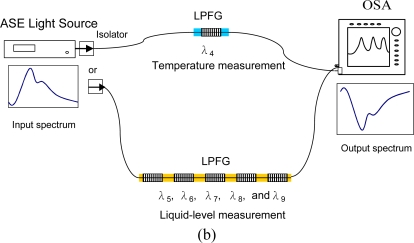
Schematic of experimental setup of **(a)** a reference dual-wavelength grating FBG sensing system for temperature and strain measurements; **(b)** an LPFG sensing system either for temperature or liquid-level measurements.

We used fiber grating sensors, FBG and LPFG sensors, as FOSs for the NDE of smart infrastructure materials. Figure 1(a) shows a schematic of the experimental setup of a reference dual-wavelength grating FBG sensing system. The FBG sensors were used to examine the temperature and strain responses of infrastructure materials. The fiber sensor was consisted of a bare grating pair (λ_1_ and λ_2_) and a packaged reference grating (λ_3_). The bare grating pair was constructed by fusion splicing two fiber Bragg gratings in cascade with different Bragg wavelengths. The three fiber Bragg gratings at wavelengths of λ_1_, λ_2_, λ_3_ were interrogated using a broadband ASE source and an OSA. A fiber coupler was used for coupling the reflected light signals of the sensor to the OSA. The reference grating was used to measure only the temperature effect. The change in the Bragg center wavelengths Δλ*_i_* of the two gratings from the changes in temperature (Δ*T_i_*) and strain (Δε*_i_*) could be obtained using Equation (1).

Figure 1(b) displays the schematic of experimental setup of an LPFG sensing system. The LPFG sensors were used to examine the temperature and liquid-level responses of infrastructure materials. The LPFG sensors were used as either a temperature sensor (wavelength of λ_4_) or a liquid-level sensor with five LPFGs in cascade with different wavelengths (wavelengths of λ_5_, λ_6_, λ_7_, λ_8_, and λ_9_, about 20-nm wavelength interval).

### 2.1. Fiber Bragg Grating Sensors

For discriminating the strain and temperature effects, the use of reference grating [7], the use of dual wavelength gratings [8], and the use of two sensors associated with different strain and temperature responses were reported [9–14]. Another approach is to use the dual wavelength technique involving writing two superimposed Bragg gratings [8], in which the responses to temperature (κ_1T_,κ_2T_) and strain (κ_1ε_,κ_2ε_) at the same location on the structure are different. The change in the Bragg center wavelengths Δλ*_i_* of the two gratings from the changes in temperature (Δ*T_i_*) and strain (Δε*_i_*) is given by the following matrix expression:

(1) $$\Delta \lambda_{i}=\kappa_{iɛ}\Delta {ɛ}_{i}+\kappa_{\mathrm{iT}}\Delta T_{i}\;i=1,2$$

where κ*_iε_* = ∂λ/∂ε*_i_* is the strain coefficient of material related to the Poisson ratio, photoelastic constant and effective refractive index, and κ*_iT_* = ∂λ/∂*T_i_* is the temperature coefficient related to the thermal expansion and thermo-optic coefficients. The above matrix can be inverted to give temperature and strain provided that the ratio of temperature responses of the two gratings is different from that of their strain responses.

We have developed a simple and low-cost FBG sensor for monitoring civil infrastructure. Figure 1a shows the configuration of the sensor and the detection system, in which the proposed sensor was connected to the output port of a fiber coupler. The three fiber Bragg gratings at wavelengths of λ_1_, λ_2_, λ_3_ were interrogated using a broadband amplified spontaneous emission (ASE) fiber source and an optical spectrum analyzer. The reference grating was used to measure only the temperature effect. The shift in Bragg wavelength λ_3_ from temperature changes is given by:

(2) $$\Delta \lambda_{3}=\kappa_{3T}\Delta T$$

**Figure 2.**
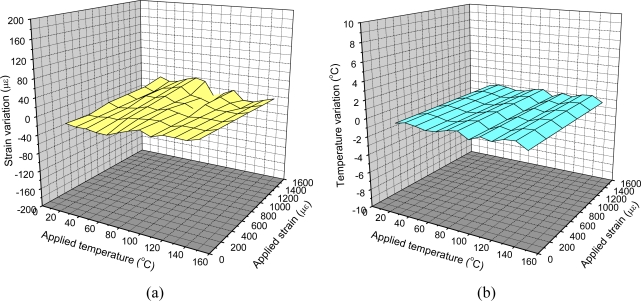
3-D Surface plot of **(a)** strain variation and **(b)** temperature variation of simultaneous strain and temperature measurements within the temperature range 30–120 °C and strain range of 0–1,500 με.

The strain coefficient of the sensor was obtained by varying the applied strain from 200 to 2,200 με while keeping the temperature constant. An excellent linear response of wavelength shifts to applied strains was found. The slope of the linear fit to the measured wavelength shifts at various strain changes was determined as the strain coefficient (pm/με) of the investigated fiber sensor (for λ_1_ and λ_2_). For the second series of tests, those sensors were kept under strain-free conditions and temperature variations from 25 °C to 80 °C. Again, an excellent linear response of wavelength shifts to applied temperature variations was shown and the slope of the linear fit to the measured wavelength shifts at various temperature changes was determined as the temperature coefficient (pm/°C) of the investigated fiber sensor for those wavelengths of λ_1_, λ_2_, λ_3_, and λ_4_. Table 1 summarizes the experimental coefficients of the optical fiber grating sensors. The measured root mean squared errors for temperature T and strain ε were estimated to be 0.13 °C and 6 με, respectively. Using the estimation of expanded uncertainty at 95% confidence level with a coverage factor of *k* = 2.205, temperature and strain measurement uncertainties of the FBG sensor have been evaluated as 2.60 °C and 32.05 με, respectively. Figure 2 shows the 3-D surface plot of strain variation and temperature variation for simultaneous strain and temperature measurements within the temperature range 30–120 °C and strain range of 0–1,500 με [15]. If temperature and strain change, the FBG sensor system or the so-called reference dual-wavelength grating presented in this paper could measure temperature and strain separately.

### 2.2. Long-Period Fiber Grating Sensors

In general, an LPFG usually has a photo-induced periodic modulation of refractive index along the core of a single-mode fiber, with a typical perturbation of Δ*n* ∼ 10^−4^, periods between 100 μm–1 mm and length of 2–4 cm. The LPFG couples light from a guided fundamental core mode (LP*_01_*) to different forward-propagating cladding modes (HE*_1m_*) in an optical fiber, which is given by phase-matching condition [16]:

(3) $$\beta_{\mathrm{core}}^{01}-\beta_{\mathrm{cladding}}^{1m}=\frac{2\pi }{\Lambda }\;\;\;\;\;\;\;m=2,3,4,....$$

where $\beta_{\mathrm{core}}^{01}$ and $\beta_{\mathrm{cladding}}^{1m}$ are propagating constants of the fundamental core mode and *m*th cladding mode, respectively, and Λ is the period of grating. The coupling of the light into the cladding region generates a series of resonant bands centered at wavelength *λ*_m_ in the transmission spectrum. The center wavelengths *λ*_m_ of an attenuation band are solutions of the following equation [16]:

(4) $$\lambda_{m}=[{\bar{n}}_{\mathrm{core}}^{01}(n_{1},n_{2},\lambda_{m})-{\vec{n}}_{\mathrm{cladding}}^{1m}(n_{2},n_{s},\lambda_{m})]\Lambda$$

where ${\bar{n}}_{\mathrm{core}}^{01}(n_{1},n_{2},\lambda_{m})$ is the effective index of the fundamental core mode at the wavelength of *λ*_m_, which is also dependent on the core refractive index *n*_1_ and cladding refractive index *n*_2_. In Equation (4) ${\vec{n}}_{\mathrm{cladding}}^{1m}(n_{2},n_{s},\lambda_{m})$ is the effective refractive of the *m*th cladding mode at the wavelength *λ*_m_, which is related to cladding refractive index *n*_2_ and the refractive index of the surrounding medium *n_s_*.

When the concentration or the refractive index of the surrounding medium changes, also ${\vec{n}}_{\mathrm{cladding}}^{1m}(n_{2},n_{s},\lambda_{m})$ changes and a shift in the central wavelength can be obtained. The cladding modes are very sensitive to change in the refractive index of the ambient (surrounding) environment.

The fabrication of LPFGs, for *λ_4_*, has been reported elsewhere [17,18]. The LPFGs, for *λ_5_* to *λ_9_*, were fabricated by the electric-arc discharge method [19] with hydrogen-free Corning SMF-28 fibers. LPFGs are especially suitable for measurements and applications when liquids or solutions undergo a change in temperature or refractive index (RI) which can be used for sensing temperatures, liquid-levels, and chloride ions [17–23].

The experimental setup for LPFGs fabrication consists of a computer-controlled electric-arc discharge associated with a translation stage, a broadband ASE fiber source and a high-resolution OSA (ANDO AQ6315A) used to *in situ* monitor the transmission loss as the grating was written. The electric-arc discharge-induced LPFGs were about 2.2–3.6 mm long and their grating periods were about 600 μm, written with a computer-controlled electric-arc discharge system. The transmission spectrum was interrogated during the writing and its characteristics such as insertion loss, resonance peak wavelength, and peak depth were analyzed after the grating was written. With suitable fabrication parameters such as electric-arc discharge power, discharge time, grating period, and scan speed, the resulting resonance wavelengths ranging from 1,200 nm to 1,600 nm with a greater than about 20 dB peak depth were obtained.

In this study, each transmission spectrum was referenced to the background spectrum of a bare fiber in air. The 3 dB bandwidth was determined by finding the peak of the ASE spectrum, and rising by 3 dB on each side. The spectral width of the ASE spectrum was determined by the separation of these two points because each has a power spectral density equal to one half the peak power spectral densities. The resonance wavelength was calculated as the average of two wavelengths determined in the 3 dB bandwidth measurements.

## 3. Materials and Experimental Setup

FBG and LPFG sensors were surface-bonded on Portland cement concrete and asphalt mixture specimens to evaluate temperature and strain measurements. The above three physical measurands are the common measurements for infrastructures or corresponding materials. The experimental study includes temperature tests, indirect tensile strain test, and liquid-level test, using both FBG and LPFG, respectively. The temperature tests focused on not only monitoring the temperature variations of the infrastructure but also assessing the performance of FOSs. An indirect tensile strain test using a packaged FBG, which could monitor and assess the strains of infrastructures, as well as a finite element modeling were conducted to compare FBG and FEM results. Liquid-level measurements were conducted using a sensor constructed by cascading five different wavelength LPFGs, which could monitor and assess the liquid-level such as reservoirs, pipelines, tanks, and channels.

### 3.1. Infrastructure Materials

**Figure 3.**
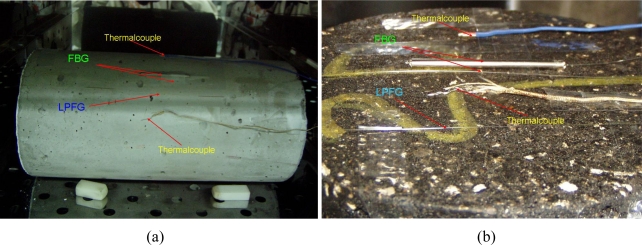
FBG and LPFG sensors bonded on the surface of infrastructure materials: **(a)** a cylindrical concrete specimen; **(b)** an asphalt mixture specimen.

Portland cement concretes and asphalt mixtures are typical infrastructure materials. The plain-concrete specimen used was 150 mm in diameter and 300 mm in height. The maximum aggregate size was 19 mm and all aggregates were crushed siliceous materials from river deposits. Type I cement was used to fabricate the cylinder specimens. The 28-day compressive strength of this concrete specimen was 24.15 kPa. Figure 3(a) shows the surface-bonded FBG and LPFG sensors on a cylindrical concrete specimen.

The asphalt specimen was 150 mm in diameter and about 116 mm in height. The maximum aggregate size was 19 mm and a PG 64-22 asphalt binder was used to fabricate the asphalt specimen. The specimen was compacted by superior performing asphalt pavement (Superpave) gyratory compactor and the aggregate structure belonged to Superpave coarse gradation (below the restricted zone) [24,25]. Both FBG and LPFG sensors were bonded on the surface of asphalt and concrete specimens as presented in Figure 3(b).

### 3.2. Temperature Tests

The temperature tests include temperature fluctuation and temperature stability tests using both FBG and LPFG sensors, respectively. Random walk coefficient (RWC) is defined as the slope of Allan variance plot (Allan deviations of temperature differences between the FOS sensor and a thermocouple versus time clusters) before Allan deviation approaches the minimum value. Bias stability (BS) is then obtained as the minimum Allan deviation and occurs at the corresponding time cluster and BS is typically described within the minimum Allan deviation with the corresponding time cluster [26–28]. These two parameters were used to evaluate the sensing performance of FOSs for all temperature tests.

#### 3.2.1. Temperature Fluctuation Tests

Temperature fluctuation tests aimed at the simulation of infrastructure materials under 24-hour temperature changes repeatedly going up and down and they could be used to assess the reliability of FOSs. Both concrete and asphalt specimen were investigated for fluctuation testing. The asphalt and concrete specimens were placed at an oven in which the temperature was employed to simulate the infrastructure temperature fluctuation conditions. Several temperature cycles were applied for a long period of time (at least one day). One temperature cycle was carried out by ramping the oven from room temperature up to a designed temperature at a rate of 3 °C/h and then rapidly down to the room temperature, normally taking 12 hours to finish one cycle. For comparison, a traditional thermocouple was also mounted near the surface of specimens during the testing, as shown in Figure 3. The temperature fluctuation tests were performed using both FBG and LPFG sensors for asphalt and concrete specimens for more than 24 hours and 48 hours, respectively.

#### 3.2.2. Temperature Stability Tests

Temperature stability tests focused on the simulation of infrastructure materials under constant temperature and they could be used to assess the stability of FOSs. Thus, the stability tests using both FBG and LPFG sensors were performed at a fixed temperature of 55 °C and 38 °C for concrete and asphalt specimens for 48 and 19 hours, respectively.

### 3.3. Indirect Tensile Strain Test

**Figure 4.**
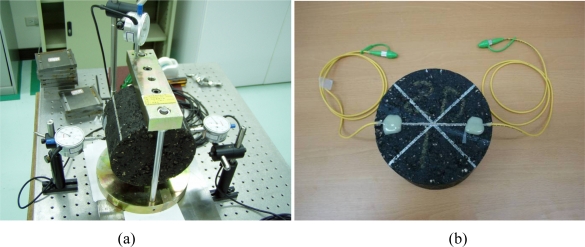
Indirect tensile test of an asphalt specimen with a packaged FBG sensor: **(a**) experimental setup; **(b)** asphalt mixture specimen.

The indirect tensile strain test for infrastructure materials was conducted using a packaged FBG sensor bonded with an epoxy glue on the surface of an asphalt specimen under the indirect tensile loading condition. Figure 4 shows the experimental setup and an asphalt specimen with a packaged FBG sensor. The strain coefficients and measurement errors of the packaged grating sensor was measured to be 1.1 pm/με and 8.6 με. The packaged FBG sensor was bonded on the surface of asphalt specimens to measure the strains with the indirect tensile loading kit (steel frames and strips). A series of static loads of 98 N, 196 N, 294 N and 392 N, were applied on the loading strip vertically. One top vertical and two-side horizontal (along the diameter) dial gauges (accuracy of 0.01 mm) were used to monitor the corresponding deformations and ensure that the relationship between applied loads and measured deformations is indirect tensile and linear-elastic. Due to the relatively lower stiffness of asphalt, it is almost impossible to bond the general-purpose strain gauge on the surface of asphalt specimens. Therefore, in this study we compared the FBG measurements with numerical simulation results.

### 3.4. Liquid-Level Test

**Figure 5.**
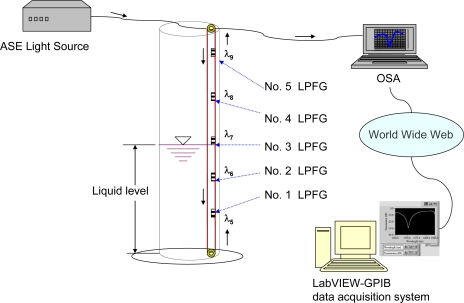
Schematic of experimental setup for liquid-level sensor constructed by cascading five different wavelength LPFGs.

Our experimental setup for the LPFG liquid-level sensing system is shown in Figure 5. A 150 mm-inside diameter, 1,200 mm-high, and hollow cylindrical storage tank having at least a 1,000 mm liquid-level capacity was used for sensing tests. We focused on a liquid-level sensor constructed by cascading five different wavelength LPFGs (wavelengths of Nos 1–5: 1,505 nm, 1,524 nm, 1,549 nm, 1,570 nm, and 1,606 nm, respectively). The liquid-level sensing tests included five levels, or five LPFGs in series with Nos. 1–5 and their corresponding fixed liquid-levels were about 230 mm, 430 mm, 640 mm, 850 mm, and 1,050 mm (from bottom to top, see Figure 5). For each liquid-level, the sensing measurements were conducted for both measurements in air and immersed in water, respectively. The wavelength shift of the LPFG has been shown to be a reliable, repeatable, and accurate measurand for several different field applications [20]. Thus, the changes of wavelengths are suggested for clearly identifying the changes and eliminating experimental errors. We performed five times liquid-level tests, in which Nos. 1–5 were all immersed in water, to identify the changes of resonance wavelength shifts for the sensor constructed by cascading five different wavelength LPFGs.

## 4. Finite Element Simulation

The FEM simulation was carried out with FEMLAB computer software and a 3-D linear static analysis was conducted to verify the feasibility of strain measurement using a FBG sensor surface-bonded on an asphalt specimen subjected to an indirect tensile loading.

### 4.1. Geometry Modeling and Meshing

**Figure 6.**
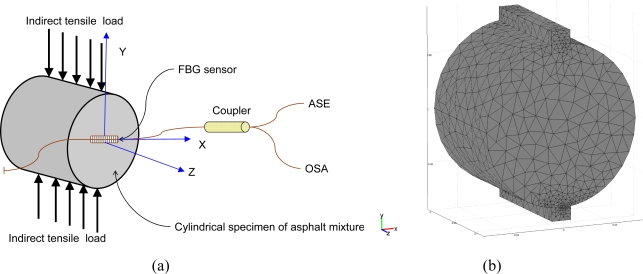
Modeling of an asphalt cylindrical specimen subjected to an indirect tensile load: **(a)** geometry plot for FBG sensing with loading strips; **(b)** normal mesh for asphalt specimen and strips.

In this work, 3-D indirect tensile loading simulation was used to model the strain measurement using a packaged FBG sensor bonded on the surface of an asphalt specimen under different indirect tensile loads. The physical model consists of an asphalt concrete cylinder specimen (150 mm in diameter and approximately 116 mm in height) and two strips (approximately 19 mm in width, 12 mm in thickness and 116 mm in height) with a FBG sensor horizontally centered along the diameter. The sensor was glued from positions at x = −40 mm to x = 40 mm. We created a 3D solid object using the Cartesian coordinate with the functions of geometry modeling and CAD tools. Figure 6a shows the geometry plot for FBG sensing with loading strips for modeling infrastructures or structures using asphalt mixtures subjected to a tensile load. The tetrahedral mesh element, Lagrange-quadratic element, was selected to create the model meshes. Three different mesh resolutions were used for the FEM simulation, corresponding to coarser, coarse, and normal model meshes that possessed a total of 4,826, 7,067, and 11,456 elements, respectively. The maximum element size default was 1/10 of the size of the geometry. The maximum element size scaling factor defaults value was 1.0. The element growth ratio was 1.4., the mesh curvature factor was 0.4, and the mesh curvature coefficient was 0.01. Figure 6(b) displays the schematic plot for a normal mesh for the asphalt specimen and loading strips. These models were used to simulate cylinders subjected to a tensile load.

Material properties of the steel loading strips are assumed to be homogeneous and isotropic. The steel loading strips possess an elastic modulus of 200 GPa, a Poisson’s ratio of 0.30, and a density of 7,850 kg/m^3^. The asphalt mixture specimen has a Poisson’s ratio of 0.35 and a density of 2,500 kg/m^3^. The elastic modulus of asphalt mixture specimen was about 0.21 GPa. The used solver was Direct (Spools), an effective direct solver for symmetric and unsymmetrical systems, based on Gaussian elimination method to solve this linear stationary problem. Table 2 summarizes the material properties of steel strips and a cylindrical asphalt mixture specimen.

### 4.2. Simulation of Cylinders Subjected to Indirect Tensile Loading

Different loading conditions were performed to determine whether the strains measured from the packaged FBG sensor agree with those obtained from FEM results. The top vertical displacements, ν, and horizontal normal strains, ε_x_, of the asphalt specimen along the x-direction were calculated using FEM method under a series of loads of 98-N, 196-N, 294-N and 392-N loading conditions. This aimed at simulating the strain measurements using the packaged FBG sensor under different indirect tensile loads.

## 5. Results and Discussion

### 5.1. Temperature Measurements

**Figure 7.**
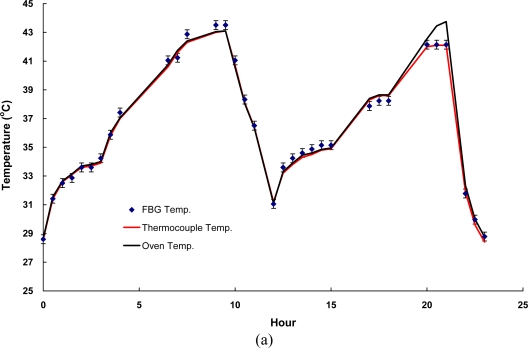

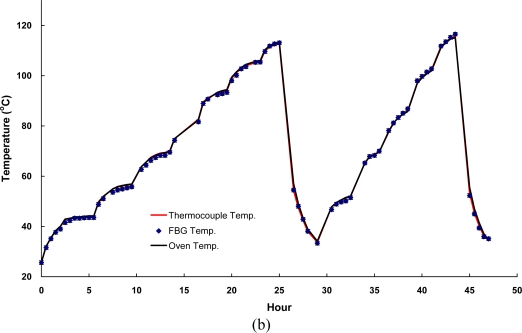
Responses of temperature fluctuation tests using FBG sensor surface-bonded on **(a)** a cylindrical asphalt mixture specimen; **(b)** a cylindrical concrete specimen.

**Figure 8.**
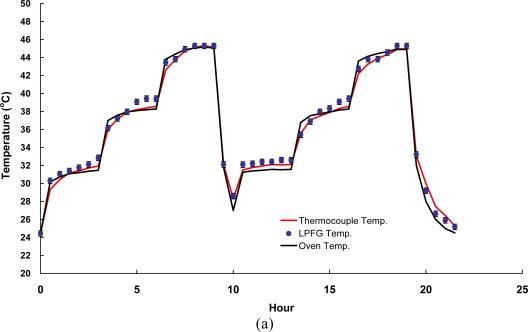

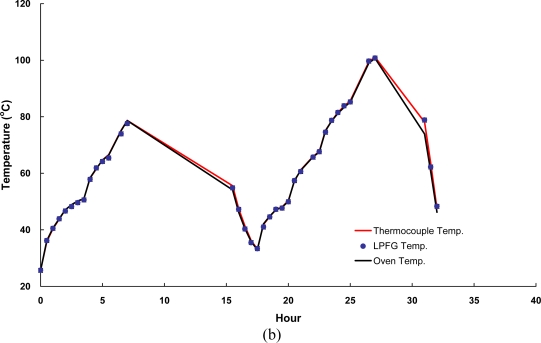
Responses of temperature fluctuation tests using LPFG sensor surface-bonded on **(a)** a cylindrical asphalt mixture specimen; **(b)** a cylindrical concrete specimen.

The responses of temperature fluctuation with time for both asphalt and concrete specimens are shown in Figures 7 and 8, respectively. The results of temperature fluctuation tests using FBG and LPFG sensors are summarized in Table 3. The random walk coefficients of temperature variations between FBG sensor and thermocouple were found as −0.9134 °C/ $\sqrt{h}$ and −1.1293 °C/ $\sqrt{h}$ for asphalt and concrete specimens, respectively. This shows that the performance of this FBG sensor is comparable with that of conventional high-resolution sensors such as thermocouple. In addition, the bias stability for temperature variations during the fluctuation tests with FBG sensor were within 0.02 °C/h with 4–5 h time cluster and 0.09 °C/h with 3–4 h time cluster for asphalt and concrete specimens, respectively.

Using the measurements from the pair of gratings, λ_1_ and λ_2_, one can determine the residual strains on the surface of the specimens at the same location, which should be near zero for these tests. In fact, a small amount of RMS strain variations were found, less than 13 με (or ∼1 °C) in all cases, as indicated in Table 3, which were all within our experimental errors.

Furthermore, the random walk coefficients of temperature variations between LPFG sensor and thermocouple were found as −1.3548 °C/ $\sqrt{h}$ and −0.9994 °C/ $\sqrt{h}$ for asphalt and concrete specimens, respectively. The bias stability for temperature variations during the fluctuation tests with LPFG sensor were within 0.03 °C/h with a 5–8 h time cluster and 0.06 °C/h with a 4–6 h time cluster for asphalt and concrete specimens, respectively. Obviously, both the FBG and LPFG sensors exhibited as the same temperature variations compared with a thermocouple.

**Figure 9.**
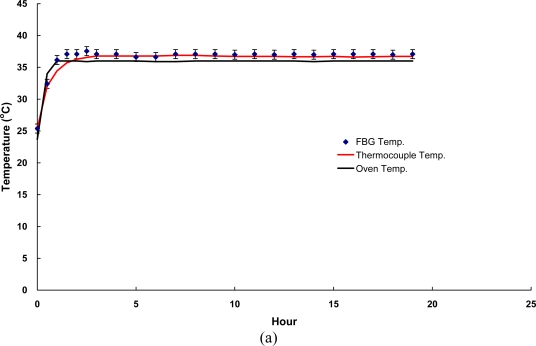

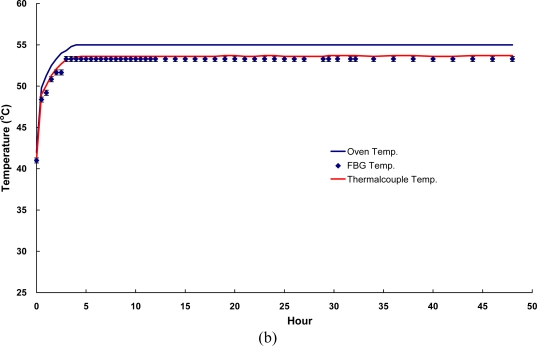
Responses of temperature stability tests using FBG sensor surface-bonded on **(a)** a cylindrical asphalt mixture specimen; **(b)** a cylindrical concrete specimen.

**Figure 10.**
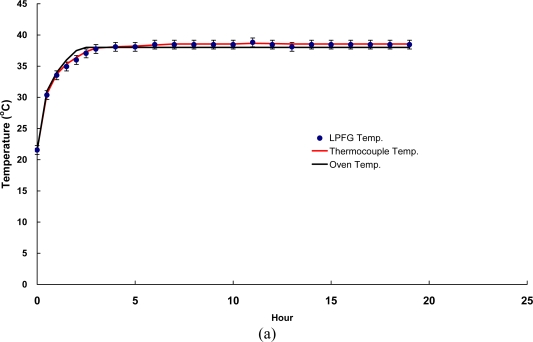

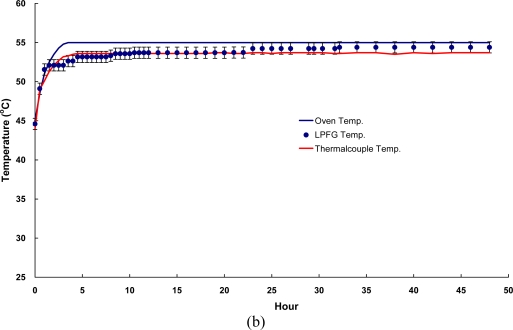
Responses of temperature stability tests using LPFG sensor surface-bonded on **(a)** a cylindrical asphalt mixture specimen; **(b)** a cylindrical concrete specimen.

The results of temperature stability tests using FBG and LPFG sensors are summarized in Table 3 and the responses of temperature stability with time for both asphalt and concrete specimens are shown in Figures 9 and 10, respectively. With the FBG sensor, the random walk coefficients of temperature variations were −0.7499 °C/ $\sqrt{h}$ and −0.9944 °C/ $\sqrt{h}$ compared with the measurements of asphalt and concrete specimens, respectively. It can be seen that the bias stability values were 0.04 °C/h with 12–15 h time cluster and 0.01 °C/h with 15–18 h time cluster for asphalt and concrete specimens. By contrast, the random walk coefficients of temperature variations between the LPFG sensor and a thermocouple were −1.0144 °C/ $\sqrt{h}$ and −0.8360 °C/ $\sqrt{h}$ compared with the measurements of asphalt and concrete specimens, respectively. Moreover, the bias stability values were 0.05 °C/h with 7–8 h time cluster and 0.03 °C/h with 15–20 h time cluster measured for asphalt and concrete specimens, respectively. Results presented here illustrate that the monitoring of temperature fluctuation and stability using FBG and LPFG sensors, was capable of measuring temperature with accuracy in the range of −0.7499 °C/ $\sqrt{h}$ to −1.3548 °C/ $\sqrt{h}$.

In addition, the bias stability for temperature variations, during the temperature fluctuation and stability tests with FBG (or LPFG) sensors were within the range of 0.01 °C/h with 15–18 h time cluster to 0.09 °C/h with 3–4 h time cluster. The excellent performance of FBG and LPFG sensors has made it possible to monitor infrastructure or structures under rugged conditions at a reasonable accuracy for a very long period of time (the fiber optic sensor is normally designed to work for at least 10 years).

### 5.2. Comparative Analysis of Indirect Tensile Strain Measurement

**Figure 11.**
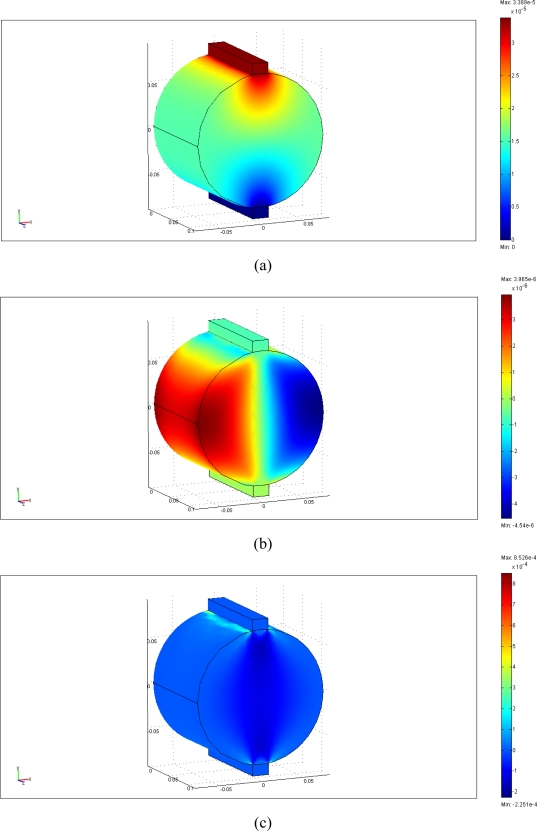
Plots of **(a)** vertical displacements; **(b)** horizontal displacements; and **(c)** horizontal normal strains of an asphalt specimen subjected to a 294-N load.

**Figure 12.**
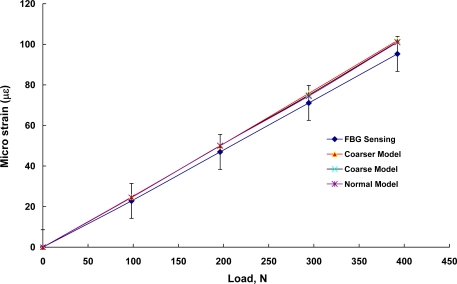
Comparison of FBG strain measurements with three different FEM modeling results.

Based on the results of FEM analysis, Figure 11 shows the plot of vertical displacements, horizontal displacements, and horizontal (diametrical) normal strains of an asphalt specimen subjected to a 294-N load, respectively. The FEM horizontal normal strains were compared with indirect tensile strains using a packaged FBG sensor. Figure 12 compares FBG experimental measurements and three different FEM mesh modeling results, which included coarser, coarse and normal mesh modeling. The measured horizontal normal strains, ε_x_, from the packaged FBG sensor agreed well with three FEM simulation predictions. In Figure 12, we used measurement error of 8.6 με as the error bar of FBG strains. It is clear that the numerical values were all located within the FBG measurements error ranges. In addition, the strain differences between the packaged FBG sensor and FEM model predictions were in the range of 4.2–6.8%. There was no significant difference among the coarser, coarse, and normal mesh models for simulating infrastructures or structures subjected to an indirect tensile loading. The study presented here demonstrates that the FBG sensor could offer the potential of simultaneous measurement of strain and temperature for monitoring infrastructure materials.

### 5.3. Liquid-Level Measurements

**Figure 13.**
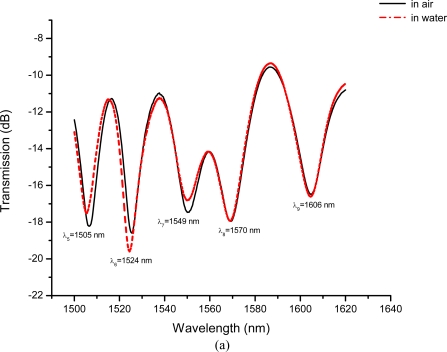

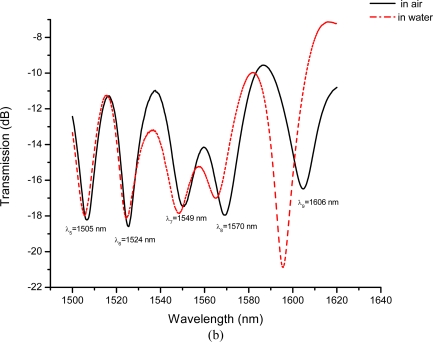
Transmission spectra of a liquid-level sensor constructed by cascading five LPFGs with different resonant wavelengths: **(a)** Nos. 1–2 immersed in water; **(b)** Nos. 1–5 immersed in water.

The liquid-level sensing tests included five liquid levels, or five LPFGs in series indexed with Nos. 15 and their corresponding liquid-level were about 230 mm, 430 mm, 640 mm, 850 mm, and 1,050 mm. Figures 13a and 13b show transmission spectra of the liquid-level sensor with LPFGs Nos. 1–2 and Nos. 1–5 immersed in water, respectively.

**Figure 14.**
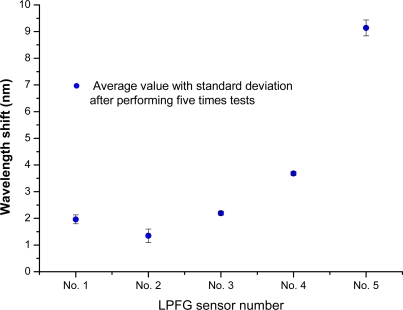
Average wavelength shifts and corresponding standard deviations by performing five times liquid-level tests (Nos. 1–5 immersed in water).

After performing liquid-level tests five times, the average values (with standard deviations) of the resonance wavelength shifts for Nos. 1–5 were about 2.0 nm (within 0.16 nm), 1.3 nm (within 0.25 nm), 2.2 nm (within 0.07 nm), 3.7 nm (within 0.07 nm), and 9.2 nm (within 0.29 nm), respectively. These results are plotted in Figure 14. The wavelength shifts of resonance wavelengths showed the LPFG-based sensor could be used to measure five different liquid levels and had at least 1050-mm liquid-level measurement capacity.

## 6. Conclusions

This paper illustrates the great potential of fundamental structural health monitoring using optical fiber grating sensors (FBG and LPFG sensors) for NDE of infrastructure materials, such as Portland cement concretes and asphalt mixtures. Three physical measurands based on different configurations of FBG and LPFG sensors were exploited, including temperature, strain, and liquid-level detection. Experimental results of two long-term stability performance parameters, random walk coefficient and bias stability, which show that the performance of a FBG (or LPFG) sensor is comparable with that of conventional high-resolution sensors such as thermocouple under rugged conditions were introduced and reported for the first time. The FEM simulation of strain measurements using a packaged FBG sensor for an asphalt specimen under indirect tensile loading conditions was shown to agree well with those experimental data at a reasonable range of errors (4.2% to 6.8%). We have also demonstrated the capacity of sensitive liquid level measurements utilizing a cascading LPFG sensor with water 105 cm-high for five different levels in a tank. For the first time, to our knowledge, based on a few simple FBG or LPFG sensor configurations, the feasibility of efficiently estimating measurement errors and monitoring long-term stability performance for simultaneous strain-temperature or liquid-level sensing of NDE for infrastructures with fiber grating sensors has been shown. Results presented here successfully demonstrate that the combination of FBG and LPFG technology has produced a surface mounted NDE based fiber sensor system capable of continuously monitoring or assessing the status of the integrity of smart structures or their components with a high level of confidence and reliability. The advantage of this type of fiber sensor is that it is more sensitive and durable than conventional sensors and is particularly well suited for on-site and remote sensing.

## Figures and Tables

**Table 1.** Experimental coefficients of optical fiber grating sensors.

**Fiber Bragg grating (FBG)**			**Long period fiber grating (LPFG)**	

**Resonance wavelength**	**Strain coefficient (pm/με)**	**Temperature coefficient, (pm/°C)**	**Resonance wavelength**	**Temperature coefficient, (pm/°C)**

λ_1_ (1,548 nm)	0.914	10.4	λ_4_^2^ (1,591 nm)	63.6
λ_2_ (1,554 nm)	0.918	12.1	λ_5_^3^ (1,505 nm)	N/A^1^
λ_3_ (1,551 nm)	N/A^1^	12.1	λ_6_ (1,524 nm)	N/A
			λ_7_ (1,549 nm)	N/A
			λ_8_ (1,570 nm)	N/A
			λ_9_ (1,606 nm)	N/A

Note:

1 N/A: not applicable;

2 λ_4_: CO_2_-induced LPFG for temperature measurement only;

3 λ_5_–λ_9_: electric-arc induced LPFGs for liquid level measurement only.

**Table 2.** Material properties of steel strips and a cylindrical asphalt mixture specimen.

Material properties	Steel strip	Asphalt mixture
Density (ρ, kg/m^3^)	7,850	2,500
Elastic modulus (E, GPa)	200	0.21
Poisson’s ratio	0.30	0.35
Mass damping parameter	1	1
Stiffness damping parameter	0.001	0.001

**Table 3.** Summary of temperature-induced testing with optical fiber grating sensors.

**Testing**	**FOS sensor**	**Material type**	**Temperature range (°C)**	**Random walk coefficient^1^ (°C/**$\sqrt{h}$**)**	**Bias stability^2^ (°C/h, with the corresponding time cluster)**	**RMSD^3^, με**	

						**δε_1_^4^**	**δε_2_^5^**

Fluctuation tests	FBG	Asphalt	25–45	−0.9134	0.02, 4–5 h	5.70	8.20
		Concrete	25–115	−1.1293	0.09, 3–4 h	12.80	11.20

	LPFG	Asphalt	25–45	−1.3548	0.03, 5–8 h	N/A^6^	N/A
		Concrete	25–100	−0.9994	0.06, 4–6 h	N/A	N/A

Stability tests	FBG	Asphalt	24–37	−0.7499	0.04, 12–15 h	N/A	N/A
		Concrete	28–55	−0.9944	0.01, 15–18 h	7.30	11.20

	LPFG	Asphalt	21–38	−1.0144	0.05, 7–8 h	N/A	N/A
		Concrete	28–55	−0.8360	0.03, 15–20 h	N/A	N/A

Notes:

1 Random walk coefficient (RWC): RWC is defined as the slope of Allan variance plot (Allan deviations of temperature differences between the FOS sensor and a thermocouple) versus time clusters.

2 Bias stability (BS): BS is obtained as the minimum Allan Deviation and occurs at the corresponding time cluster and it typically is described within the minimum Allan deviation with the corresponding time cluster.

3 RMSD: the root mean square deviation (RMSD) of measurements.

4 δε_1_: back-calculated micro strain variation for λ_1_

5 δε_2_: back-calculated micro strain variation for λ_2_.

6 N/A: not applicable
